# Supplementary material for: Fast and accurate joint inference of coancestry parameters for populations and/or individuals
Source: PLoS Genet. 2023 Jan 19;19(1):e1010054. doi: 10.1371/journal.pgen.1010054 (PMC9888729; doi:10.1371/journal.pgen.1010054)
Supplement: S1 Text — (PDF) [file pgen.1010054.s001.pdf]

S1 Text Proofs.

Lemma 1. *Let $F_1, ..., F_K$ be random variables satisfying for $k = 1, 2, \ldots, K{-}1$:*

$$\mathbb{E}\left[F_{k+1}|F_k = p_k\right] = p_k, \qquad \mathbb{V}\mathrm{ar}\left[F_{k+1}|F_k = p_k\right] = \theta_k p_k(1{-}p_k)\ .$$

Then

$$\begin{aligned} \mathbb{E}\left[F_K|F_1 = p_1\right] &= p_1, \\ \mathbb{V}\mathrm{ar}\left[F_K|F_1 = p_1\right] &= \left[1{-}\prod_{k=1}^{K-1}(1{-}\theta_k)\right] p_1(1{-}p_1)\ . \end{aligned}$$

The proof is by induction. The case $k = 1$ is given as an assumption in the statement of the Lemma. Assuming that the required property is satisfied for $k = K{-}1$, and writing g for the conditional probability density function of the F_k, we can express $\mathbb{V}\mathrm{ar}\left[F_K|F_1{=}p_1\right]$ as:

$$\begin{aligned} &= \int (p_K{-}p_1)^2\, g\left(p_K\,|p_1\right) \\ &= \int \left[(p_K{-}p_{K-1})^2 + (p_{K-1}{-}p_1)^2 + 2\,(p_K{-}p_{K-1})\,(p_{K-1}{-}p_1)\right] g\left(p_K\,|p_{K-1}\right) g\left(p_{K-1}\,|p_1\right) \\ &= \int \left[\theta_{K,K-1}p_{K-1}(1{-}p_{K-1}) + (p_{K-1} - p_1)^2\right] g\left(p_{K-1}\,|p_1\right) \\ &= \theta_{K,K-1}\left[p_1{-}\left[1{-}\prod_{k=1}^{K-2}(1{-}\theta_k)\right] p_1(1{-}p_1){-}p_1^2\right] + \left[1{-}\prod_{k=1}^{K-2}(1{-}\theta_k)\right] p_1(1{-}p_1) \\ &= p_1(1{-}p_1)\left\{\theta_{K,K-1}\left[1{-}\left[1{-}\prod_{k=1}^{K-2}(1{-}\theta_k)\right]\right] + 1{-}\prod_{k=1}^{K-2}(1{-}\theta_k)\right\} \\ &= p_1(1{-}p_1)\left\{\theta_{K,K-1}\prod_{k=1}^{K-2}(1{-}\theta_k) + 1{-}\prod_{k=1}^{K-2}(1{-}\theta_k)\right\} \\ &= p_1(1{-}p_1)\left\{1{-}\prod_{k=1}^{K-1}(1{-}\theta_k)\right\}. \end{aligned}$$

Now recall the definition of $F^W_{ST}(kk')$ in (2), which can be expressed as:

$$F^W_{ST}(kk') \quad = \quad \mathbb{C}\mathrm{or}\left[x_k, y_{k'}\right] = \frac{\mathbb{C}\mathrm{ov}\left[p_k, p_{k'}\right]}{p(1{-}p)}$$

and, recalling that M denotes the most recent common ancestor of k and k',

$$\begin{aligned} \mathbb{C}\mathrm{ov}\left[p_k, p_{k'}\right] &= \int (p_k{-}\mathbb{E}\left[p_k\right])\,(p_{k'}{-}\mathbb{E}\left[p_{k'}\right])\, g\left(p_k, p_{k'}\,|p\right) \\ &= \int (p_k{-}p)\,(p_{k'}{-}p)\, g\left(p_k, p_{k'}\,|p_M, p\right) g\left(p_M\,|p\right) \\ &= \int (p_M{-}p)^2\, g\left(p_M\,|p\right) = \mathbb{V}\mathrm{ar}\left[p_M|p\right] \end{aligned}$$

which, together with Lemma 1, establishes (12), the first equality in Theorem 1.

Now recall the definition of $F^H_{ST}(kk')$ at (3). Focusing first on the denominator,

$$\mathbb{P}\left[x_k \neq y_k\right] \quad = \quad \int \left(x_k(1{-}y_k) + y_k(1{-}x_k)\right) g\left(x_k, y_k\,|p\right)$$

$$
\begin{aligned}
&= 2\int p_k(1-p_k)g\,(p_k\,|p\,) \\
&= 2\left\{\mathbb{E}\,[p_k|p]\,-\mathbb{V}\mathrm{ar}\,[p_k|p] - [\mathbb{E}\,[p_k|p]]^2\right\} \\
&= 2\left\{f-\left(1-\prod_{q\in\mathcal{P}(k)}(1-\theta_q)\right)p(1-p)-p^2\right\} \\
&= 2p(1-p)\prod_{q\in\mathcal{P}(k)}(1-\theta_q)
\end{aligned}
$$

where the last equality comes from Lemma 1. Similarly,

$$
\mathbb{P}\,[x_k \neq x_{k'}] \quad = \quad 2p(1-p)\prod_{q\in\mathcal{Q}(kk')}(1-\theta_q)\ .
$$

Therefore,

$$
\begin{aligned}
F_{ST}^{H}(kk') &= 1-\frac{1}{2}\frac{\prod_{q\in\mathcal{P}(k)}(1-\theta_q)+\prod_{q\in\mathcal{P}(k')}(1-\theta_q)}{\prod_{q\in\mathcal{Q}(kk')}(1-\theta_q)} \\
&= 1-\frac{1}{2}\left\{\prod_{q\in\mathcal{R}(kk')}(1-\theta_q)+\prod_{q\in\mathcal{R}(k'k)}(1-\theta_q)\right\}. \\
&= \frac{1}{2}\left\{\mathbb{C}\mathrm{or}\,[x_k,x_k|p_M]+\mathbb{C}\mathrm{or}\,[x_{k'},x_{k'}|p_M]\right\}.
\end{aligned}
$$

which completes the proof of Theorem 1

The proof of Proposition 1 follows in the same way. $\mathbb{E}\,[N_k]$ can be derived from the computation of $\mathbb{P}\,[x_k \neq y_k]$, and the derivation of $\mathbb{E}\,[D_{kk'}]$ corresponds to the computation of $\mathbb{P}\,[x_k \neq x_{k'}]$.
