# Supplementary material for: Fast and accurate joint inference of coancestry parameters for populations and/or individuals
Source: PLoS Genet. 2023 Jan 19;19(1):e1010054. doi: 10.1371/journal.pgen.1010054 (PMC9888729; doi:10.1371/journal.pgen.1010054)
Supplement: S2 Text — (PDF) [file pgen.1010054.s002.pdf]

## S2 Text Details of the tree-based inference algorithm

Here we use the notations of Table 1, now including subscripts such as $T$ to identify the current tree. A tree-like population model $T$ is characterized by the set of paths to each sampled population from ancestral Population 0, $\{\mathcal{P}_T(k),\ k \in \{A, ..., K\}\}$, together with $\theta$ values for each tree branch.

### Phase 1: Clustering.

We propose an ascending clustering strategy to obtain an approximately optimal $\widehat{T}$. We introduce

$$\mathcal{C}_1(T) = \{K : \mathrm{par}_T(K) = 0\}$$

the set of all (sampled or ancestral) populations whose parent population is Population 0. The ascending algorithm, also illustrated on a 4 population example in Fig. 3, works as follows:

Step 1 *Initialization*: Set $j = 1$ and let $T_1$ denote the independent-descent population model (Fig. 1), so that

$$\mathcal{C}_1(T_1) = \{A, ..., K\}\,;$$

Step 2 *Merging*: Increment $j$. Consider each pair $\{c_1, c_2\} \in \mathcal{C}_1(T_{j-1})$ and add an intermediate ancestral population $j$ in tree $T_{j-1}$ to create a new tree $T_{c_1c_2} = T_{j-1} \cup \{j\}$ such that $\mathrm{par}_{T_{c_1c_2}}(c_1) = j$, $\mathrm{par}_{T_{c_1c_2}}(c_2) = j$, and $\mathrm{par}_{T_{c_1c_2}}(j) = 1$. Infer the $\beta$ for each $T_{c_1c_2}$ using (11), and then set $T_j = T_{c_1^*c_2^*}$ where

$$\{c_1^*, c_2^*\} = \underset{\{c_1,c_2\}}{\arg\min} \sum_{k,k'} \left( \log S_{kk'} - \sum_{q \in \mathcal{R}_{T_{c_1c_2}}(kk')} \widehat{\beta}_q \right)^2 .$$

Step 3 *Stopping*: If $j = K-1$ set $\widehat{T} = T_j$ and stop, else go to step 2.

### Phase 2: Improvement.

We attempt to update $\widehat{T}$ by choosing a tip node $k$ in random order, and consider relocating its parent to each branch of the current tree, choosing the branch that minimizes $\xi$.
