# Supplementary material for: Fast and accurate joint inference of coancestry parameters for populations and/or individuals
Source: PLoS Genet. 2023 Jan 19;19(1):e1010054. doi: 10.1371/journal.pgen.1010054 (PMC9888729; doi:10.1371/journal.pgen.1010054)
Supplement: S3 Text — (PDF) [file pgen.1010054.s003.pdf]

S3 Text Joint estimation of inbreeding and coancestry effects.

The coancestry parameters θ_k measure divergence between populations due to multiple evolutionary processes, including inbreeding. Here, we seek to estimate inbreeding coefficients in addition to the θ_k, allowing us to separate out the contribution of inbreeding from other processes. For individual i of population k, we write (x_{i1}, x_{i2}) for the genotype at a given locus, where the allele indicators x_{i1} and x_{i2} has the same first two moments as x_k, introduced above (2), but now we additionally assume that

$$\mathbb{C}\text{ov}\left[x_{i1}, x_{i2}|p_k\right] = \phi_i p_k(1{-}p_k)$$

and we refer to ϕ_i as the inbreeding coefficient of individual i. We now use n_k to denote the number of diploid individuals in population k, so that the number of alleles is $2n_k$. We also define

$$\bar{\phi}_k = \frac{1}{n_k}\sum_{i=1}^{n_k}\phi_i$$

the average inbreeding coefficient in population k.

Recall from its definition at (9) that $S_{kk'} \propto N_k/D_{kk'}$. While $D_{kk'}$ is unaffected by inbreeding, N_k now involves terms of the form $x_{i1}x_{i2}$. We first derive an expression for $\mathbb{E}\left[N_k\right]$ when the parent of Population k is the ancestral population. Noting that the expectations are conditional on the ancestral allele frequency p, we have $\mathbb{E}\left[N_k\right] = \mathbb{E}\left[\hat{p}_k(1{-}\hat{p}_k)\right] = p - \mathbb{E}\left[\hat{p}_k^2\right]$ and so we require an expression for $\mathbb{E}\left[\hat{p}_k^2\right]$. Introducing the notation g for probability density function,

$$\begin{aligned}
4n_k^2\mathbb{E}\left[\hat{p}^2\right] &= \sum_{i=1}^{n_k}\sum_{j=1}^{n_k}\sum_{u=1}^{2}\sum_{v=1}^{2}\mathbb{E}\left[x_{iu}x_{jv}\right] \\
&= \sum_{i\neq j}\sum_{u=1}^{2}\sum_{v=1}^{2}\mathbb{E}\left[x_{iu}x_{jv}\right] + \sum_{i}\sum_{u\neq v}\mathbb{E}\left[x_{iu}x_{iv}\right] + \sum_{i}\sum_{u}\mathbb{E}\left[x_{iu}^2\right] \\
&= 4n_k(n_k{-}1)\mathbb{E}\left[x_{11}x_{22}\right] + 2\sum_i\mathbb{E}\left[x_{i1}x_{i2}\right] + 2n_k\mathbb{E}\left[x_{11}^2\right] \\
&= 4n_k(n_k{-}1)[\theta_k p(1{-}p){+}p^2] + 2\sum_i\int[\phi_i p_k(1{-}p_k){+}p_k^2]g(p_k) + 2n_k p \\
&= 4n_k(n_k{-}1)[\theta_k p(1{-}p){+}p^2] + 2\sum_i\phi_i(1{-}\theta_k)p(1{-}p) + 2n_k[\theta_k p(1{-}p){+}p^2] + 2n_k p \\
&= 2n_k\left\{2(n_k{-}1)[\theta_k p(1{-}p){+}p^2] + \bar{\phi}_k(1{-}\theta_k)p(1{-}p) + [\theta_k p(1{-}p){+}p^2] + p\right\}.
\end{aligned}$$

Then,

$$\begin{aligned}
\mathbb{E}\left[N_k\right] &= \frac{1}{2n_k}\left\{2n_k p - 2(n_k{-}1)[\theta_k p(1{-}p){+}p^2] - \bar{\phi}_k(1{-}\theta_k)p(1{-}p) - [\theta_k p(1{-}p){+}p^2] - p\right\} \\
&= \frac{1}{2n_k}\left\{(2n_k{-}1)(1{-}\theta_k)p(1{-}p) - \bar{\phi}_k(1{-}\theta_k)p(1{-}p)\right\} \\
&= \frac{2n_k{-}1}{2n_k}(1{-}\theta_k)p(1{-}p)\left(1 - \frac{\bar{\phi}_k}{2n_k{-}1}\right).
\end{aligned}$$

In the general case that the parent of Population k may not be the ancestral population, a similar derivation leads to

$$\mathbb{E}\left[S_{kk'}\right] \approx \frac{\mathbb{E}\left[N_k\right]}{\mathbb{E}\left[D_{kk'}\right]} = \frac{2n_k{-}1}{2n_k}\left(1 - \frac{\bar{\phi}_k}{2n_k{-}1}\right)\prod_{q\in\mathcal{R}(kk')}(1{-}\theta_q)\ . \tag{1}$$

Neglecting $1/2n_k$ and the bias in the ratio of expectations, we then obtain

$$\mathbb{E}\left[\log(S_{kk'})\right] = \mu_k + \sum_{q \in \mathcal{R}(kk')} \beta_q \quad \text{where} \quad \mu_k = \log\left(1 - \frac{\bar{\phi}_k}{2n_k - 1}\right), \quad \beta_q = \log(1-\theta_q) \ .$$

If the average inbreeding coefficients $\bar{\phi}_k$ are known, then our tree-based estimation procedure can be applied by simply replacing $\log(S_{kk'})$ with $\log(S_{kk'}) - \mu_k$ in (11). If the μ_k are unknown, we see from (1) that θ_k and $\bar{\phi}_k$ cannot be separately estimated by our procedure. This matches intuition that more inbreeding leads to a higher variance in allele frequency, which can be fitted by increasing either θ_k or $\bar{\phi}_k$.

However, using

$$\mathbb{P}\left[x_{i1} \neq x_{i2}\right] = 2p(1-p)(1-\theta_k)(1-\phi_i)$$

we can minimise the squared difference between expected and empirical heterozygosity proportions for each individual, in conjunction with the optimisation (11), to achieve joint inference of individual ϕ_i and the θ_k. The complexity of such an algorithm would be $\mathcal{O}(n^3)$ rather than $\mathcal{O}(K^3)$, where n is the total sample size. This computational cost would be prohibitive for large n, but practical strategies may be available for example focussing on separating out inbreeding and coancestry effect sizes only in some populations or individuals.
