# Supplementary material for: Fast and accurate joint inference of coancestry parameters for populations and/or individuals
Source: PLoS Genet. 2023 Jan 19;19(1):e1010054. doi: 10.1371/journal.pgen.1010054 (PMC9888729; doi:10.1371/journal.pgen.1010054)
Supplement: S4 Text — (PDF) [file pgen.1010054.s004.pdf]

S4 Text F_{ST}^{H} for the simulation model with admixture.

We modify the simulation model of Fig. 4 so that the parent of Population C is drawn from both Populations 1 and 3, with fraction α (the admixture proportion) coming from Population 1. Thus $\alpha = 0$ corresponds to the simulation model of Fig. 4.

Based on Definition (3) the computation of the different coefficients F_{ST}^{H} requires the derivation of probabilities

$$P_{kk'} = \mathbb{P}\left[x_k \neq y_{k'}\right], \quad k, k' \in \{A, B, C, D, E\} \; .$$

Compared with the reference case $\alpha = 0$, most of these quantities remain unchanged or require only minor modifications, the exceptions being the cases with $k = C$. We provide the full derivation of $F_{ST}^{H}(BC)$, which from (3) involves P_{BB}, P_{BC} and P_{CC}. The remaining F_{ST}^{H} values are obtained similarly and we give only brief details.

$$\begin{aligned}
P_{BC}/2 &= \mathbb{P}\left[x_B \neq y_C\right]/2 = \mathbb{E}\left[x_B(1-y_C) + y_C(1-x_B)\right]/2 \\
&= p - \mathbb{E}\left[x_B y_C\right] = p - \int p_B p_C g(p_B, p_C) \\
&= p - \int \left\{\alpha p_1^2 + (1-\alpha) p_1 p_3\right\} g(p_1, p_3) \\
&= p - \alpha\left[\theta_1 p(1-p) + p^2)\right] - (1-\alpha)p^2 \\
&= \alpha(1-\theta_1)p(1-p) + (1-\alpha)p(1-p) \; .
\end{aligned}$$

This expression can be summarized as follows: with probability α the allele drawn in population C comes from population 1, in which case the most recent common ancestor population is 1, and with probability $1-\alpha$ the allele comes from population 3, and the most recent common ancestor population is 0. Similarly,

$$\begin{aligned}
P_{BB}/2 = p - \mathbb{E}\left[x_B y_B\right] &= p - \int p_B^2 g(p_B) \\
&= p - \int \left[\theta_B p_1(1-p_1) + p_1^2\right] g(p_1) \\
&= p - \int \left[\theta_B p_1 + (1-\theta_B) p_1^2\right] g(p_1) \\
&= (1-\theta_B)(p - \theta_1 p(1-p) - p^2) \\
&= (1-\theta_B)(1-\theta_1)p(1-p) \; .
\end{aligned}$$

This is the same as in the reference setting, since population B is unaffected by admixture in this case. Lastly:

$$\begin{aligned}
\mathbb{E}\left[x_C y_C\right] &= \int p_C^2 g(p_C) \\
&= \int \theta_C \left[\alpha p_1 + (1-\alpha)p_3\right]\left[1 - \alpha p_1 - (1-\alpha)p_3\right] + \left[\alpha p_1 + (1-\alpha)p_3\right]^2 g(p_1, p_3) \\
&= \alpha^2 \int \left[\theta_C p_1(1-p_1) + p_1^2\right] \; g(p_1) + (1-\alpha)^2 \int \left[\theta_C p_3(1-p_3) + p_3^2\right] \; g(p_3) \\
&\quad + \alpha(1-\alpha)\theta_C \int \left[p_1(1-p_3) + p_3(1-p_1)\right] \; g(p_3, p_1) + 2\alpha(1-\alpha) \int p_1 p_3 \; g(p_3, p_1) \\
&= \alpha^2 \left[\theta_C p + (1-\theta_C)\left[\theta_1 p(1-p) + p^2\right]\right] + \alpha(1-\alpha)\theta_C p(1-p) + 2\alpha(1-\alpha)p^2 \\
&\quad + (1-\alpha)^2 \left[\theta_C p + (1-\theta_C)\theta_3 p + (1-\theta_C)(1-\theta_3)\left[\theta_2 p(1-p) + p^2\right]\right]
\end{aligned}$$

which leads to

$$P_{CC} = 2p(1-p)(1-\theta_C)\left\{\alpha^2(1-\theta_1) + 2\alpha(1-\alpha) + (1-\alpha)^2(1-\theta_3)(1-\theta_2)\right\} \; .$$

Gathering the 3 results above, we obtain

$$F_{ST}^{H}(BC) = 1 - \frac{(1-\theta_C)\left\{\alpha^2(1-\theta_1) + (1-\alpha)^2(1-\theta_3)(1-\theta_2) + 2\alpha(1-\alpha)\right\} + (1-\theta_B)(1-\theta_1)}{2(\alpha(1-\theta_1) + (1-\alpha))} .$$

In the case $\alpha = 0$ we obtain the corresponding expression from Theorem 1.

Derivation of $F_{ST}^{H}(AC)$

$$\begin{aligned}
\mathbb{E}\left[x_A y_C\right] &= \int p_1(\alpha p_1 + (1-\alpha))p_2 g(p_1, p_2) = \alpha\left[\theta_1 p(1-p) + p^2\right] + (1-\alpha)p^2 \\
P_{AC} &= 2p(1-p)\left[\alpha(1-\theta_1) + (1-\alpha)\right] \\
P_{AA} &= 2p(1-p)(1-\theta_A)(1-\theta_1)
\end{aligned}$$

leading to

$$F_{ST}^{H}(AC) = 1 - \frac{(1-\theta_C)\left\{\alpha^2(1-\theta_1) + (1-\alpha)^2(1-\theta_3)(1-\theta_2) + 2\alpha(1-\alpha)\right\} + (1-\theta_A)(1-\theta_1)}{2(\alpha(1-\theta_1) + (1-\alpha))} .$$

Derivation of $F_{ST}^{H}(CD)$

$$\begin{aligned}
\mathbb{E}\left[x_C y_D\right] &= \alpha p^2 + (1-\alpha)\left[\theta_3 p + (1-\theta_3)\left[\theta_2 p(1-p) + p^2\right]\right] \\
P_{CD} &= 2p(1-p)\left[\alpha + (1-\alpha)(1-\theta_3)(1-\theta_2)\right] \\
P_{DD} &= 2p(1-p)(1-\theta_D)(1-\theta_3)(1-\theta_2)
\end{aligned}$$

and

$$F_{ST}^{H}(CD) = 1 - \frac{(1-\theta_C)\left\{\alpha^2(1-\theta_1) + (1-\alpha)^2(1-\theta_3)(1-\theta_2) + 2\alpha(1-\alpha)\right\} + (1-\theta_D)(1-\theta_3)(1-\theta_2)}{2(\alpha + (1-\alpha)(1-\theta_3)(1-\theta_2))} .$$

Derivation of $F_{ST}^{H}(CE)$

$$\begin{aligned}
\mathbb{E}\left[x_C y_E\right] &= \alpha p^2 + (1-\alpha)\left[\theta_2 p(1-p) + p^2\right] \\
P_{CE} &= 2p(1-p)\left[\alpha + (1-\alpha)(1-\theta_2)\right] \\
P_{EE} &= 2p(1-p)(1-\theta_E)(1-\theta_2)
\end{aligned}$$

and

$$F_{ST}^{H}(CE) = 1 - \frac{(1-\theta_C)\left\{\alpha^2(1-\theta_1) + (1-\alpha)^2(1-\theta_3)(1-\theta_2) + 2\alpha(1-\alpha)\right\} + (1-\theta_E)(1-\theta_2)}{2(\alpha + (1-\alpha)(1-\theta_2))} .$$
