# Supplementary material for: Fast and accurate joint inference of coancestry parameters for populations and/or individuals
Source: PLoS Genet. 2023 Jan 19;19(1):e1010054. doi: 10.1371/journal.pgen.1010054 (PMC9888729; doi:10.1371/journal.pgen.1010054)

S1 Fig Inferred tree showing coancestry among the 15 individuals and 3 population samples. Similar to Fig 7B except that the 5 individuals from CHB, IBS and MSL samples have been pooled.

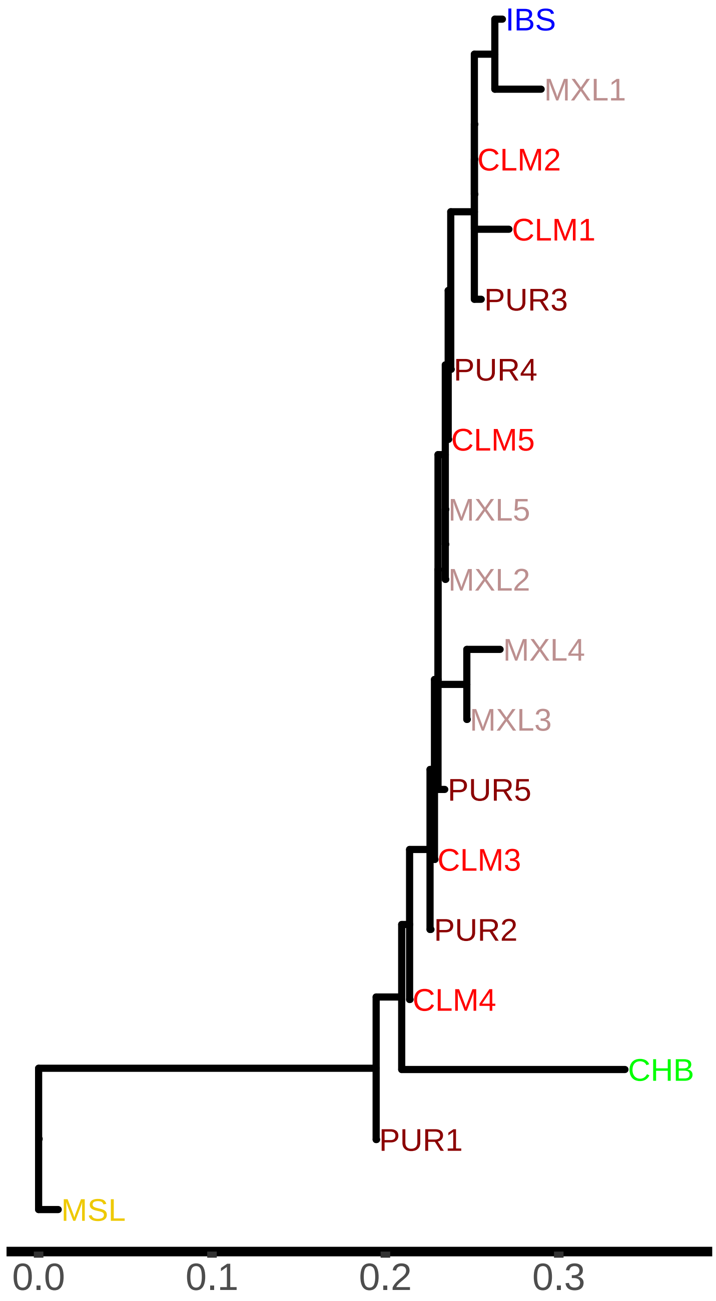

Supplement: S1 Fig — (PDF) [file pgen.1010054.s005.pdf]
