# Supplementary material for: Fast and accurate joint inference of coancestry parameters for populations and/or individuals
Source: PLoS Genet. 2023 Jan 19;19(1):e1010054. doi: 10.1371/journal.pgen.1010054 (PMC9888729; doi:10.1371/journal.pgen.1010054)
Supplement: S1 Table — (PDF) [file pgen.1010054.s006.pdf]

S1 Table 1000 Genomes Project identifiers for the 5 individuals from each of 6 populations in the individual analyses.

Population	Individual ID
CLM	HG01112 HG01119 HG01122 HG01125 HG01131
MXL	NA19648 NA19652 NA19661 NA19681 NA19684
PUR	HG01161 HG01164 HG01168 HG01173 HG01177
CHB	NA18525 NA18528 NA18531 NA18533 NA18535
IBS	HG01500 HG01503 HG01506 HG01509 HG01512
MSL	HG03052 HG03058 HG03063 HG03069 HG03079
