# Supplementary material for: Fast and accurate joint inference of coancestry parameters for populations and/or individuals
Source: PLoS Genet. 2023 Jan 19;19(1):e1010054. doi: 10.1371/journal.pgen.1010054 (PMC9888729; doi:10.1371/journal.pgen.1010054)
Supplement: Attachment — Submitted filename: AEComments.pdf [file pgen.1010054.s007.pdf]

This paper is likely to be on interest, especially as Balding has given several treatments of F_{ST} estimation in the past. Both reviewers are also expert in this field, and the authors need to consider all their points carefully. I am in full agreement with all the comments.

Although I am flattered by being linked to Wright by the F^W notation, I would prefer to see the authors refer to the much more recent Weir and Goudet (Genetics 206:2085-2103, 2017) and Zhang, Goudet and Weir (Heredity 128:1-10, 2022).

I agree with Reviewer 1 on the need to discuss the consequences of assuming independent populations (not assumed by Weir and Goudet), and with Reviewer 2 on discussing pairs of populations rather that individual-specific values.

I look forward to the authors' detailed responses and revised manuscript.
