# Supplementary material for: Fast and accurate joint inference of coancestry parameters for populations and/or individuals
Source: PLoS Genet. 2023 Jan 19;19(1):e1010054. doi: 10.1371/journal.pgen.1010054 (PMC9888729; doi:10.1371/journal.pgen.1010054)
Supplement: Attachment — Submitted filename: MaryHuardBalding.pdf [file pgen.1010054.s009.pdf]

PGENETICS-D-22-00076R1

Reviewer 1 Please emphasize the strong assumption about limited gene flow.

Reviewer 2 There doesn't need to be distinction made between F_{ST}^W and F_{ST}^H. They can both be cast as (functions of) ibd probabilities.

Associate Editor Comments P3, Figure 1 Legend. Weir and Hill [3] explicitly did not assume independent-descent populations.

P3, lines 62-63. Drawing information from all sampled populations was integral to Weir and Hill [3] and to Weir and Goudet [8].

P3. Equations 1. This is the model of Weir and Cockerham [2], but zero covariance not used in [3] or [8].

P4, Equations 2. Weir-Hill[3], page 377, did not require a zero covariance of indicators for alleles from different populations.

P4,Line 78. Not clear why x and y have different symbols here: it is not because $k \neq k'$. Need to explain for the numerator in Equation (3) that x, y are for distinct random alleles from the same population.

P4, Line 85. Maybe useful to show $\Pr(x_k \neq y_k) = 2p(1-p)(1-\theta_k)$, $\Pr(x_k \neq y_{k'}) = 2p(1-p)$. There is no need to regard F_{ST}^W and F_{ST}^H as being based on different concepts: they can both follow from θ_k being defined as the ibd probability for a random pair of distinct alleles from population k.

P4, Equation 3. Not clear why F_{ST}^H is shown only for two populations. Equation 4 could be generalized to multiple populations, and F_{ST}^H recognized as the Weir and Goudet [8] estimator expressed with allele miss-matches rather than allele matches.

P5, Line 117. Is $A(k)$ the immediate parent of k, or just ancestral to k?

P6, Line 130. Maybe useful to say that $\{a, b\}$ is for two values a and b, but (a, b) and $[a, b]$ are for intervals with boundaries a, b.

P6, Line 141. What about the unknown p's?

P17, above Line 422. What is α?

P19, Lines 451-452. Why not simplify this to show the variance

$$\mathrm{Var}(\hat{p}_k) \quad = \quad p(1-p)\left[\theta_k + \frac{1}{2n_k}(1-\theta_k)(1+\bar{\phi}_k)\right]$$

and say that θ_k can be regarded as an ibd probability, but the within-population inbreeding coefficient $\bar{\phi}_k$ (the population-specific "F_{IS}") is not a probability but a correlation that can be negative. If F_k is the average inbreeding coefficient for population k (an ibd probability) then $\bar{\phi}_k = (F_k - \theta_k)/(1-\theta_k)$ and the variance of $\hat{p}_k$ has the term $(1 + F_k - 2\theta_k)/(2n_k)$ as in our writings.
