# Supplementary material for: Fast and accurate joint inference of coancestry parameters for populations and/or individuals
Source: PLoS Genet. 2023 Jan 19;19(1):e1010054. doi: 10.1371/journal.pgen.1010054 (PMC9888729; doi:10.1371/journal.pgen.1010054)
Supplement: Attachment — Submitted filename: MaryHuardBalding.pdf [file pgen.1010054.s011.pdf]

PGENETICS-D-22-00076R2

Reviewers Please consider the reviewers' comments.

Associate Editor Comments The authors still include incorrect statements about cited publications [3] and [8]:

1. Line 8: [2] and [3] used the method of moments estimation, not sum-of-squares estimation.
2. Line 81: The statement "Following Weir-Hill [3] we define $F_{ST}^{W} = \mathrm{Cor}[x_k, y_{k'}|p) = 0$ if $k \neq k'$" is not correct. The whole thrust of the Weir-Hill paper was to allow correlated allele frequencies among populations.
3. Line 355-357: "[8] note that 'it is not possible to find estimates for each [Fst]... when sampled populations have correlated sample allele frequencies.'" By changing θ to F_{ST} the present authors have given an inaccurate quote and a misrepresentation of the whole thrust of the Weir-Goudet paper. Weir and Goudet were clear to distinguish between probabilities of identity by descent (θ) and F_{ST} quantities (β). The rest of the quoted paragraph includes "it is possible to rank β values, and, we expect these to have the same ranking as their expected values θ" which changes the meaning of what the present authors imply. The actual expected values of the β's are functions of different θ's as shown in Table 3 of [8]. Weir and Goudet gave explicit closed-form estimators for F_{ST} and these estimators allow for the presence of inbreeding.

Some minor points include

1. Line 136: "For the proof, see" is missing a citation.
2. Line 173: "$p \notin (0.0, 0.99)$" in spite of authors' claim in their response to reviews not to use (...) notation.
3. Line 284: "Greater divergence of non-AFR populations may reflect an out-of-Africa bottleneck." seems weak when compared to Line 304 "consistent with the origins of modern humans in Africa."
4. Line 333: "and hence FST estimates" should state which FST estimates are being used.
