# Supplementary material for: Fast and accurate joint inference of coancestry parameters for populations and/or individuals
Source: PLoS Genet. 2023 Jan 19;19(1):e1010054. doi: 10.1371/journal.pgen.1010054 (PMC9888729; doi:10.1371/journal.pgen.1010054)
Supplement: Attachment — Submitted filename: Response2reviewers.odt [file pgen.1010054.s008.odt]

Response to reviewers:
Many thanks to the reviewers for your positive and constructive comments.  They led us to do a substantial amount of new work in the revised version, exploring extensions of our approach to inbreeding and admixed populations.  Also in response to reviewer comments we have changed the structure of the paper, now we present the key theoretical result in Results which required other changes to maintain the logical flow, and we have changed notation and terminology as requested.  As a result of such major changes it is not practical to submit a version highlighting the changes made; these are so numerous that it would not be helpful to reviewers.  We describe the main changes in our responses below, with line numbers referring to the new version of the manuscript.
Reviewer comments are in italics followed by our responses.
We thank the reviewers for their comments, and in particular these positive comments not requiring a response:
R1: I think this paper is very interesting and ultimately may be very useful.
R2: The most insightful contribution of this study is the clarification of the relationship between the correlation-based estimator of Weir and colleagues (Weir and Cockerham 1984; Weir and Hill 2002) and the IBD formulation of Hudson et al. (1992).
Comments with responses:
Reviewer #1:
A great deal more care needs to be taken in terms of clarity of mathematical definitions and more standard use of terms.
We’ve adopted all the specific suggestions made by the reviewer, and taken the opportunity to insert more explanations, described further below.
... strictly applies to a very, very small subset of applications of Fs, excluding even the human history to which the method is applied to here. ... applied to simulations of populations that experience migration, so that the approach can be ground-truthed  ... to determine whether the method gets sensible answers in this more realistic context.
Strictly, the method is applicable to all populations, it does not seek to reconstruct history but find a best-fitting tree structure to describe the actual genetic variation. However we accept that for many sets of populations, there may not be a tree structure that accurately represents the actual genetic variation.  This is likely to be the case when the population history includes substantial effects from admixture.  We now include results from a new simulation with admixture between highly-diverged populations (described at L187-191), and we make the first steps towards explicitly incorporating admixture into our approach by showing in this example that the definitions and computational formulae that we derive in Theorem 1 extend naturally to admixture graphs (Appendix S4).  Doing this systematically for a general class of admixture graphs, and generalising our least-squares tree-fitting approach to such graphs, is beyond the scope of this paper and will require a substantial further research effort which we plan to seek funding to pursue, as is now briefly mentioned in the Discussion (L360-365)
28: It is more usual to refer to “tips” rather than “leaves”
We have removed many references to leaves and changed others to “tip nodes”.
48-52: … make clear what you mean by shared and unshared, and how these relate to the different FST’s.
We’ve added explanation (L52-59) and given an example illustrated with colour coding in Fig 2.
67-68: The standard term in population genetic is allele frequency not allele fraction. I’d suggest changing the term used
We’ve made this change.
67-68: Please, please don’t use f for this quantity; p is a good alternative.
Done.
71: Define divergence, especially in terms of theta. A key parameter here (theta) is essentially undefined.
We have removed the reference to divergence here, and in other places but retained it in a few places where it carries its ordinary English meaning and requires no definition.  We now introduce theta at the start of the Results section (L226-228), pointing out that it corresponds to the Weir/Wright Fst in the independent-descent model.
Table 1: ... the \ notation – spell out the intent here. Discuss the meaning of Q, P and R in the text
This is now done in the table caption.
104-106: I didn’t follow this paragraph
It has been removed.
Eq 11: Explain what this equation is doing, and justify this choice for beta hat.
We have changed the organisation and wording to include some explanation of the statistic S and motivation for the least-squares formula (L127-137).
147: It seems out of place to have this line about FST_H when the topic of the preceding is FST_W.
We’ve rewritten extensively to balance coverage F^H and F^W
177: Define MAF
It is defined on L16.
Simulation section (174-190): This section seems wholly inadequate to describe to the reader how they may recreate the procedure followed here.
It is a simple simulation model and all key details were there, but we have reworded and expanded the description for increased clarity.
188: Says “See Methods”, but this is part of the Methods section. Where is this intended to point?
Thanks for spotting this error, now fixed.
218: Giving the run time is not meaningful with info about the computer infrastructure used.
We have clarified, but since the time is only around 10 minutes on a desktop computer, precise details are not important.
Table 4: Perhaps give bias of FST_W hat for comparison?
We did not refer to any FST_W hat in the paper, and indeed we are not aware of any alternative FST_W estimator with which to compare our novel estimator.
While considering this comment we came to the view that rather than reporting bias for F^W and RMSE for F^H, it is better to report RMSE for both.  In the case of F^H we can compare tree and pairwise estimators, for F^W there is no such comparison but we can compare with values for F^H and observe that estimation of F^W tends to be a little less precise, as expected since it measures more ancient correlations.
Table 5: My main impression from Table 5 is that these RMSE ratios are all very close to 1, and the effect of the new procedure is small.
We agree, but our new estimation procedure does much more and remains very fast.  We added a row to Table 4 to report the average improvement, and inserted a brief discussion of this point (L253-256).
... very useful to know how dependent the method is on the choice of populations to be included ... populations that are more closely related than average ... could get quite different answers than if the samples are more independently taken.
“closely related” populations will tend to produce smaller theta values than “independent” populations, but neither poses any particular problem for the method which can be applied to samples from any set of populations.
Reviewer #2:
The authors’ formulation does not consider inbreeding … the extent to which the authors’ framework relates to the much more general formulation of Weir and Goudet,
Thanks for this suggestions, we have now introduced Appendix S3 dealing with inbreeding.
... some of the properties of the new estimators are duly mentioned in the material and methods but are not revisited in the discussion.
Thanks for this comment which drew our attention to the fact that the derivation of formulae for F^W and F^H  generalised to tree-structured populations should be in the Results section, as it is a key result of the paper, as the reviewer has noted.  We also moved there the comment about the effects of addition or removal of populations, to give them more prominence.
… when evaluating the performance of the new population-specific estimator of Fst, the authors only present results for three of the five scenarios (S1, S3, and S6).
We only presented results for 3 scenarios for our estimator of F^W because (as noted in response to R1 above) we do not have an existing estimator for comparison.  In the case of our estimator of F^H, we can compare with a popular pairwise estimator and so it is of interest to present more detailed results, using all 6 simulation scenarios.
