# Supplementary material for: Fast and accurate joint inference of coancestry parameters for populations and/or individuals
Source: PLoS Genet. 2023 Jan 19;19(1):e1010054. doi: 10.1371/journal.pgen.1010054 (PMC9888729; doi:10.1371/journal.pgen.1010054)
Supplement: Attachment — Submitted filename: Response2reviewers.odt [file pgen.1010054.s010.odt]

Response to reviewers and associate editor:
Many thanks to the reviewers for your further helpful comments which are copied here in italics followed by our responses in red.
Reviewer #1:
... the authors think carefully about much of the work including admixture to include here ... having some of the work on admixture here may steal some thunder from the future paper.
Thanks for this thoughtful comment. The admixture results were derived and written up in response to previous reviewer comments and so they are less mature than other parts of the paper.  We feel that the results here are sufficiently preliminary that they will not detract from any future publication, but instead are intended to highlight the limitations of our tree-based approach as raised by the reviewer.  Therefore, on reflection, we are inclined to keep the admixture results.
the abstract ought to make clear that this work is suited for populations whose history can be described accurately by a tree structure. It should be clear to the reader that the work has not yet encompassed situations where there is persistent and regularly occurring gene flow ...
We have reworded the abstract to help address this point, particularly the first two sentences.  Also in the discussion we state “The inferred tree … may not accurately reconstruct ... because there is no explicit role for admixture” and we have now added “However, in the presence of admixture it may be the case that no tree can accurately capture the genetic structure” (L359 – 360).
Reviewer #2:
The new version is very clearly written and I could only identify few additional and relatively minor issues: ... the Material and Methods sections .. equal attention to both estimators, the simulation results are completely focused on F^H; ... there is no reference in the text to F^W results …
This is not quite true, F^W results are discussed in the text at L233 – 239, L248 – 252, L266, L279 – 285 and L324 – 327.  
… need to mention that the precision of the F^W is lower than that of F^H.
We have expanded the previous sentence to expand this point, now L248 – 251.
...(Simulation Study) they have to mention that they will focus on F^H and only present limited results for F^W and they should also explain why.
We have added a sentence at L251 – 252 clarifying that, while F^H is useful for pairs of populations, F^W is valuable as a measure of divergence of a single individual/population, which is reflected in the subsequent results.
... several assumptions ... are mentioned under Material and Methods but I think they should also be briefly mentioned in the discussion as many interested users may not read the methodological details but still need to be aware of the assumptions being made when they use the new estimators.
L242 – 245 have been moved from Material and Methods to Results to make these points more prominent.
Figure 2: ... the two populations referred to at the end of the caption should be CD instead of BC.
Oops, now fixed, thanks.
AE comments:
Figure 1 Legend. Weir and Hill [3] explicitly did not assume independent-descent populations.
We removed the reference to [3] in the legend.
P3, lines 62-63. Drawing information from all sampled populations was integral to Weir and
Hill [3] and to Weir and Goudet [8].
We have added these two references.
P3. Equations 1. This is the model of Weir and Cockerham [2], but zero covariance not used in
[3] or [8].
Ref [2] is now cited here (L 75), [3] and [8] are cited in the Discussion.
P4, Equations 2. Weir-Hill [3], page 377, did not require a zero covariance of indicators for alleles
from different populations.
We now explicitly mention that the second equality comes from Equation 1, so that the reference to [3] is only relevant to the first equality.
P4, Line 78. Not clear why x and y have different symbols here: it is not because k != k′. Need
to explain for the numerator in Equation (3) that x, y are for distinct random alleles from the same
population.
Done (L 86).
P4, Line 85. Maybe useful to show Pr(xk != yk) = 2p(1−p)(1−θk), Pr(xk != yk′ ) = 2p(1−p).
There is no need to regard FSTW and FSTH as being based on different concepts: they can both follow from θk being defined as the ibd probability for a random pair of distinct alleles from population k.
Our theoretical framework does not rely on IBD as a basis for definition. IBD-based definitions are versatile, allowing the (experimented) user to choose the reference population, but this comes to the price of making this choice a priori, and the implications of different choices are not easy for users to comprehend. Our preferred approach is to start from concrete and intuitive definitions based on  correlation and mismatch probabilities of alleles, that are standard in the literature, and then to derive closed form expressions for the Fst that can then be connected with the IBD framework based on our specified reference populations, which provides the reader with additional options for interpretation. 

P4, Equation 3. Not clear why FSTH is shown only for two populations. Equation 4 could be
generalized to multiple populations, and FSTH recognized as the Weir and Goudet [8] estimator
expressed with allele miss-matches rather than allele matches.
The more general version is not needed for our development, but is now noted at L 87.
P5, Line 117. Is A(k) the immediate parent of k, or just ancestral to k?
A(k) stands for the immediate parent of k, as defined at L118.
P6, Line 130. Maybe useful to say that {a, b} is for two values a and b, but (a, b) and [a, b] are
for intervals with boundaries a, b.
We now define the [a,b] notation at first use L78, we avoid use of (a,b) for intervals and we believe that the set  notation {a,b} is sufficiently standard that it does not require definition.
P6, Line 141. What about the unknown p’s?
The unknown p’s play no role in definition of the beta coefficients nor in computing the Skk’ terms.
P17, above Line 422. What is α?
Thanks for pointing out this typo, now fixed 
P19, Lines 451-452. Why not simplify this to show the variance and say that θk can be regarded as an ibd probability, but the within-population inbreeding coefficient ̄φk (the population-specific “FIS ”) is not a probability but a correlation that can be negative. If Fk is the average inbreeding coefficient for population k (an ibd probability) then φk = (Fk − θk)/(1 − θk ) and the variance of ˆpk has the term (1 + Fk − 2θk)/(2nk ) as in our writings.
As discussed above, we prefer to start with moments of allele frequencies as basic quantities, which leads here to expressions that appear lengthy but the steps are straightforward and intuitive. This is not a full treatment of inbreeding but only an exploration, within an appendix, of what is estimable within our approach.
