# Supplementary material for: Fast and accurate joint inference of coancestry parameters for populations and/or individuals
Source: PLoS Genet. 2023 Jan 19;19(1):e1010054. doi: 10.1371/journal.pgen.1010054 (PMC9888729; doi:10.1371/journal.pgen.1010054)
Supplement: Attachment — Submitted filename: Answer to the editor.docx [file pgen.1010054.s012.docx]

**Associate Editor Comments**

*Line 8: [2] and [3] used the method of moments estimation, not sum-of-squares estimation.*

We have reworded this paragraph to give more emphasis to method-of-moments type approaches (and the rewording has changed reference numbers), but we have retained the sums of squares description for WC84 because that is how it is often discussed, there isn’t a big distinction in the biallelic case and the presentation is in terms of “components of variance. Further, WG17 refer to “the sums of squares used by WC84”.

*Line 81: The statement “Following Weir-Hill [3] we define FST = Cor[xk, ykt |p) = 0 if k /= k' ” is not correct. The whole thrust of the Weir-Hill paper was to allow correlated allele frequencies among populations.*

We had changed the accompanying text to describe the different justifications for the two equalities. For further clarity, we have broken up the formula into two parts, there is only one equality now in eq (2).

*Line 355-357: “[8] note that ‘it is not possible to find estimates for each [Fst]... when sampled populations have correlated sample allele frequencies.’ ” By changing θ to FST the present authors have given an inaccurate quote and a misrepresentation of the whole thrust of the Weir-Goudet paper. Weir and Goudet were clear to distinguish between probabilities of identity by descent (θ) and FST quantities (β).*

Our replacement of θ with Fst in our quote from WG17 is valid if Fst is understood as the parameter defined in the present work (and in much previous work, see further below). However since WG17 defined Fst to be different from θ, we agree that our use could be misleading. We have now reworded avoiding the symbol θ and referring instead to ``allele correlation parameter”. Although WG17 consider the definition of Fst based on a reference set derived from the observed populations, which brings certain advantages, for our work it is preferable to adhere to the definition of Fst as an allele correlation parameter (for two populations, equivalent to the θ defined in eq (1) of WG17). This allelic correlation definition of Fst was also explicit WC84 (p1358) and WH02.

*The rest of the quoted paragraph includes “it is possible to rank β values, and, we expect these to have the same ranking as their expected values θ” which changes the meaning of what the present authors imply.*

We now briefly summarise this point (L358-9) it does not change our fundamental point that our tree structure allows us to obtain explicit expressions for all of our parameters – and consequently for the allele correlation parameter - in a context where there is correlations between sampled allele frequencies, whereas WG17 note that this is not possible in a general setting.

*The actual expected values of the β’s are functions of different θ’s as shown in Table 3 of [8]. Weir and Goudet gave explicit closed-form estimators for FST and these estimators allow for the presence of inbreeding.*

We agree that WG17 provide closed-form estimators, whereas we provide closed-form expressions for the θ parameters (i.e. the allele correlation parameters), as well as estimators for these quantities.

*Some minor points include*

*Line 136: “For the proof, see” is missing a citation.*

We’re not sure what problem arose, “S1 Appendix” appears in my copy of the submitted version, it seems that it was not visible on the editor’s copy.

*Line 173: “p ∈/ (0.0, 0.99)” in spite of authors’ claim in their response to reviews not to use (...) notation.*

We now defined both [] and () at L78.

*Line 284: “Greater divergence of non-AFR populations may reflect an out-of-Africa bottle-neck.” seems weak when compared to Line 304 “consistent with the origins of modern humans in Africa.”*

We don’t believe that any change is needed.

*Line 333: “and hence FST estimates” should state which FST estimates are being used.*

θ underlies all our Fst estimates, we have reworded to replace Fst wiith “Fst ^W and Fst^H”.
